# “Blanket rules just don’t work”: Qualitative exploration of the relative value of child and adult quality-adjusted life year (QALY) gains for health technology assessment

**DOI:** 10.1017/S0266462325000194

**Published:** 2025-03-28

**Authors:** Marcus Sellars, Joanna Coast, Emily Lancsar, Cam Donaldson, Stacy M. Carter

**Affiliations:** 1 Department of Health Economics Wellbeing and Society, National Centre for Epidemiology and Population Health, College of Health & Medicine, The Australian National University, Canberra, Australia; 2 Centre for Quality and Patient Safety Research (QPS), Institute of Health Transformation, School of Nursing and Midwifery, Deakin University, Burwood, Australia; 3 Health Economics Bristol, Population Health Sciences, Bristol Medical School, University of Bristol, Bristol, UK; 4 Yunus Centre for Social Business & Health, Glasgow Caledonian University, Glasgow, UK; 5 Australian Centre for Health Engagement, Evidence and Values (ACHEEV), School of Health and Society, Faculty of the Arts, Humanities and Social Sciences, University of Wollongong, Keiraville, Wollongong, New South Wales, Australia

**Keywords:** Adolescent health services, Australia, decision making, health technology assessment, quality-adjusted life years

## Abstract

**Objectives:**

Effective allocation of scarce healthcare resources involves complex ethical and technical evaluations, with decision makers sometimes utilizing a societal perspective in health technology assessment (HTA). This study aimed to explore societal perspectives on healthcare resource allocation within Australia’s HTA framework, focusing on the valuation of health gains for children and young people (CYP) compared to adults.

**Methods:**

In-depth, semistructured interviews were conducted with ten young people (aged 15–17) and twenty adults between October 2021 and April 2022. Participants were purposively sampled for diverse characteristics and completed an online information survey prior to the interviews, introducing relevant concepts. Interviews were analyzed using inductive coding, categorization, and constant comparison.

**Results:**

Participants expressed nuanced perspectives on HTA processes, generally opposing numeric weighting and preferring a deliberative approach based on committee judgment. Although most participants acknowledged some moral relevance of CYP status in HTA, opinions varied on its operationalization. A sizable minority, including those with extensive health system experience, did not view CYP status as morally relevant, though some noted specific service gaps for CYP (e.g., mental health care, pain management). Participants identified a spectrum of factors, both person-centered and intervention related, that often surpassed the relevance of CYP status, including addressing severity, unmet needs, prevention, and early intervention, with an emphasis on Aboriginal and Torres Strait Islander communities.

**Conclusion:**

Our findings highlight the inherent challenges in navigating the complexities of HTA and the critical need for HTA frameworks to be adaptable and inclusive, effectively integrating societal preferences to enhance healthcare policy’s equity and responsiveness.

## Introduction

Health technology assessment (HTA) evaluates the safety, effectiveness, and cost-effectiveness of health technologies for adults and children and young people (CYP) (1–3). Cost-effectiveness analysis (CEA) estimates the additional cost per quality-adjusted life year (QALY) gained, using the incremental cost-effectiveness ratio (ICER) (4). Thresholds vary by country (5), with some using standardized thresholds, whereas others use more flexible ones. In Australia, the Pharmaceutical Benefits Advisory Committee (PBAC) and the Medical Services Advisory Committee (MSAC) assess an interventions’ safety, effectiveness, and cost-effectiveness to make recommendations on “value for money” without fixed thresholds (6–8).

HTA has an ethical dimension (9–11), often implicit rather than explicit (6;7). Although reliant on technical assessments, it also addresses prioritization in resource allocation, making decision making inherently normative. Public values and preferences vary (12), including what is evaluated and on what basis (e.g., maximizing benefit, considering equity) (13;14). Integrating these preferences is complex (15), requiring accurate measurement to form QALYs (16). Methods to enhance this process include preference elicitation studies to determine how QALYs should be weighted, influencing HTA calculations and guiding deliberations (17).

Beyond preference elicitation, strategies to make the ethics of HTA more explicit include integrating broader societal values through multicriteria decision analysis (MCDA) and deliberative frameworks (18). These approaches address dimensions such as equity, family impacts, and long-term societal benefits, complementing traditional methods like preference elicitation for QALYs; recent work has identified a number of these societal values in HTA processes internationally (19).

Some HTA bodies, such as those in the Netherlands, Norway, the United Kingdom, and Sweden, employ numeric-based weighting to address health disparities (5). In contrast, others evaluate health gains from a common baseline, integrating societal values or other difficult-to-quantify information case by case (20). “Numeric weighting” refers to assigning specific numerical values, whereas “deliberative weighting” involves committee judgment for the systematic consideration and prioritization without assigning specific numerical values.

Age in healthcare resource allocation has been extensively explored, but public views on the relative value of a QALY for children compared to adults remain unclear. No decision-making bodies numerically differentiate health gains by age, though severity-based weighting may impact age groups differently, as seen in Norway and the Netherlands (21). For instance, younger children may be prioritized for treatments with long-term developmental benefits due to their potential for more life-years gained. Conversely, older adults may experience reduced prioritization in frameworks emphasizing QALYs gained, raising ethical concerns about age-based equity. In Australia, the PBAC may consider age implicitly for equity or ethical assumptions, evaluated case by case rather than weighed numerically (7).

Qualitative studies on age prioritization rarely contextualize age within HTA processes (22) and often exclude of CYP perspectives. Many report that some participants make exceptionalist judgments about CYP (22), arguing that their health gains should be weighted more heavily than adult health gains in HTA, often using equity-based arguments, such as fair innings (23–25), and highlighting the need to address a perceived lack of voice for CYP in society (26).

Other studies reveal public concerns: about arbitrary age cutoffs, or that factors other than age are more important, including disease burden, benefit potential, and societal contribution (24;27–29). Some advocate for equal treatment of all patients, irrespective of age (27). In a recent UK interview study, adult participants, not asked about the specific context of decision making in HTA, suggested a nuanced approach, advocating for decisions on a case-by-case basis over uniform processes (24).

Key research gaps remain in understanding whether society values health gains differently for CYP compared to adults. No study has directly engaged CYP about whether CYP health gains should be assessed differently to adult health gains. Although substantial efforts have been made in quantitative studies to understand distributional weighting of QALYs that include age (13;30–32), few studies have explicitly engaged the public in qualitative discussions about potential health gain weightings for CYP within the context of HTA (23). Including the perspectives of younger people is essential for creating more effective and equitable health policies, ensuring the unique needs and perspectives of those directly impacted by pediatric healthcare policies are considered, particularly as adolescence is a critical developmental period requiring tailored health interventions (33).

To address these gaps, we aimed to explore in-depth both adult and CYP perspectives on the valuation of CYP’s health gains relative to those of adults within the context of HTA processes in Australia. Our analysis focused on the weighting of health gains, the moral relevance of CYP status in HTA decision making, and the other underlying principles that should guide HTA decision making.

## Methods

### Approach

We conducted a qualitative study, using in-depth semistructured interviews with Australian young people and adults, employing reflexive thematic analysis (34;35). Guided by a constructivist perspective, we recognized knowledge as co-constructed and shaped by interpretive processes when exploring participants’ experiences. We maintained a reflective stance, acknowledging our diverse disciplinary backgrounds (psychology, ethics, and economics) could influence data interpretation. The study is reported according to the Consolidated Criteria for Reporting Qualitative Research (COREQ) (36).

### Sampling and recruitment

Young people (aged 15–17) and adults (aged 18+) from the Australian general public were recruited using targeted social media advertisements in collaboration with the Population Health Exchange at the Australian National University. Advertisements on Facebook and Twitter linked to an online screening survey. We purposively sampled participants to ensure diversity across a range of demographic characteristics, including location, education level (adults), current health conditions, and parental status (adults). For parental status, we sought to include participants who were currently parents of CYP, parents of adult children, and those who had never been parents. We restricted recruitment to ages 15 and older due to practical, ethical, and developmental considerations. The topic’s complexity required abstract reasoning, and the feasibility of conducting online qualitative research of this complexity with younger participants during COVID-19 was uncertain.

Three campaigns were run chronologically to achieve the final sample: (1) general population of adults (aged 18+) and young people (aged 15–17); (2) young males; and (3) adult males. The latter two campaigns targeted groups under-represented in the first campaign.

### Data collection

An interview guide included in Online Supporting Information 1 was developed based on (i) a systematic literature review of social values of QALYs (22), (ii) semistructured interviews conducted with committee members of the PBAC and MSAC regarding current decision-making processes (8) and their views on social values of QALYs, and (iii) team discussions. Participants’ age, gender, state or territory, postcode, general health state, and current disability were obtained in the screening survey. For adults, we also collected information on child dependents, their general health status, and any current disabilities. Socio-Economic Indexes for Areas (SEIFA) deciles (37) were calculated using participants’ postcodes.

**Figure 1.**
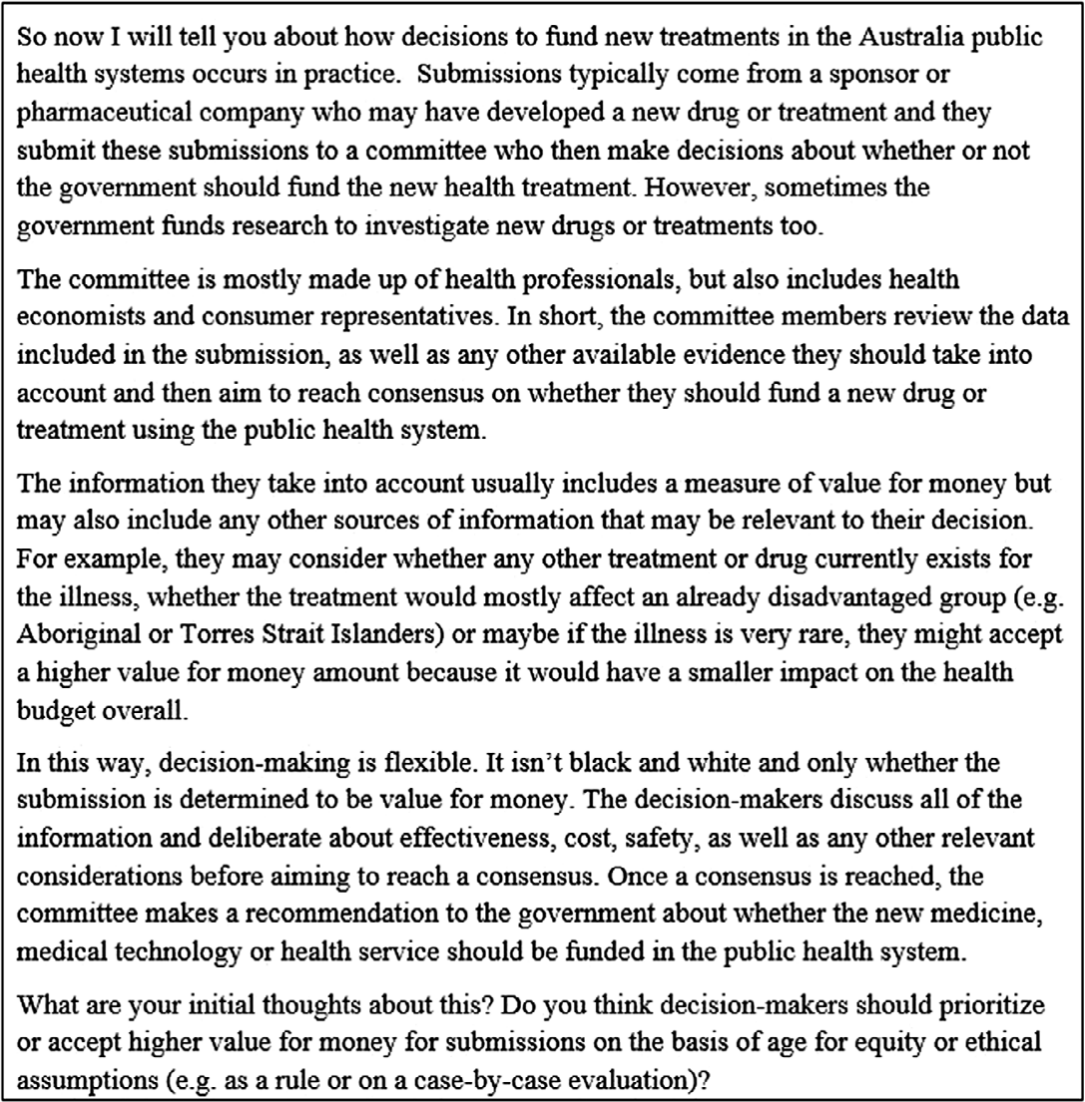
Excerpt from interview guide: Description of current decision-making processes in Australia based on findings from interviews with committee members involved in PBAC and MSAC processes (8).

Participants completed a preinterview survey (Online Supporting Information 2) introducing the topics for discussion, including government funding decisions for medicines and treatments, and arguments for prioritizing health care for CYP despite lower “value for money” compared to adults. During the interview, after discussing these topics, the interviewer described Australia’s HTA processes further explore participants’ beliefs and values (Figure 1).

MS conducted semistructured interviews with participants during September 2021 and February 2022 on the video conferencing platform Zoom. Interviews were digitally audio-recorded and transcribed verbatim. Participants were asked if they wanted to receive a one-page summary of their interview to provide additional feedback (member checking) and 13 said yes.

### Data analysis

The analysis was led by MS, who coded the transcripts and wrote detailed memos. Regular analytical discussions were held with the research team throughout the process to refine interpretations and explore emerging patterns of meaning.

Initially, three research team members (MS, SC, and JC) independently open coded two early transcripts. MS then worked within NVivo Plus (version 12; QSR International) to organize, code, and search data. Themes were developed inductively by coding the transcripts to identify key concepts in the data and develop a revised coding structure. Through constant comparison of concepts across the transcripts and discussions within the research team (38), key positions regarding CYP status, weighting, and HTA processes were identified. MS, with assistance from SC and JC, developed a new coding structure and recoded the transcripts. Data were extracted by theme, providing a basis for the analysis. Text search queries and framework matrices helped identify relationships and patterns within the data. The final thematic structure was shaped through iterative engagement and discussion among the research team, with CD and EL contributing to the interpretation and final analytical narrative.

Participant quotes illustrating each theme are included in the text. For each quote, information is given about the person speaking in terms of their unique identifier, along with their gender, age, self-described health state, and their experience of the health system (described as minimal, moderate, or extensive). For adult participants, information about whether they had children is also provided. Quotes are presented verbatim, with ellipses used to denote missing speech; “umm,” “err,” “you know,” “I mean,” “like,” and repeats of words that do not add to meaning are removed without use of ellipsis.

### Ethical considerations

The Australian National University Human Ethics Review Committee approved our study (Protocol: 2021/124). Informed consent was obtained before each interview.

## Results

Between October 2021 and April 2022, 282 people opened the screening survey, 124 provided a contact email agreeing to be interviewed, 51 people were invited to schedule an interview, and 35 people responded. Thirty people (ten young people, aged 15–17) completed the preinterview information survey and proceeded to interview (see Table 1 for participant characteristics).

**Table 1.** Characteristics of young people (*n* = 10) and adult participants (*n* = 20)

	Young people *n* (%)	Adults *n* (%)	Total *n* (%)	Population (15 years and older) (%)
Gender				
Female	6 (60.0)	8 (40.0)	14 (46.7)	51.1
Male	3 (30.0)	11 (55.0)	14 (46.7)	48.9
Nonbinary	1 (10.0)	1 (5.0)	2 (6.7)	–
Mean age ± standard deviation	16 ± 1	44 ± 20	35 ± 21	–
Location of residence				
Australian Capital Territory	–	3 (15.0)	3 (10.0)	1.8
New South Wales	1 (10.0)	3 (15.0)	4 (13.3)	32.0
Northern Territory	–	–	–	0.9
Queensland	2 (20.0)	2 (10.0)	4 (13.3)	20.3
South Australia	1 (10.0)	2 (10.0)	3 (10.0)	7.0
Tasmania	–	2 (10.0)	2 (6.7)	2.2
Victoria	4 (40.0)	5 (25.0)	9 (30.0)	25.6
Western Australia	2 (20.0	3 (15.0)	5 (16.7)	10.5
Socio-Economic Indexes for Areas (SEIFA), Australia decile				
1–3 (Low)	1 (10.0)	2 (10.0)	3 (10.0)	30.0
4–7 (Medium)	1 (10.0)	4 (20.0)	5 (16.7)	40.0
8–10 (High)	8 (80.0)	14 (70.0)	22 (73.3)	30.0
Has children (adults)				
Yes	–	10 (50.0)	10 (50.0)	–
Has children who are currently dependent on them (adults)				
Yes	–	7 (70)	7 (70)	53
Self-reported health in general				
Excellent	1 (10.0)	1 (5.0)	2 (6.7)	–
Very good	3 (30.0)	8 (40.0)	11 (36.7)	–
Good	2 (20.0)	6 (30.0)	8 (26.7)	–
Fair	3 (30.0)	5 (25.0)	8 (26.7)	–
Poor	1 (10.0)	–	1 (3.3)	–
Self-reported disability, health condition or injury that has lasted, or is likely to last, 6 months or more which restricts with everyday activities				
Yes	3 (30.0)	4 (20.0)	7 (23.3)	–
No	6 (60.0)	13 (65.0)	19 (63.3)	–
Maybe	1 (10.0)	3 (15.0)	4 (13.3)	–
Parent-reported child disability, health condition or injury that has lasted, or is likely to last, 6 months or more which restricts their everyday activities				
Yes	–	1 (5.0)	1 (5.0)	–
No	–	6 (30.0)	6 (30.0)	–
Maybe	–	3 (15.0)	3 (15.0)	–

*Note:* Population data sourced from the Australian Bureau of Statistics (44).

### Overview

The results reported next focus on the factors on which participants place a social value and their beliefs regarding weighting health factors in HTA, with a particular focus on the underlying principles that should guide HTA decision making. Four main themes were identified:

1. Inherent tensions in developing consistent positions
2. Weighting of QALYs in HTA submissions
3. Moral relevance of CYP status in priority setting
4. Governing HTA decision making

**Figure 2.**
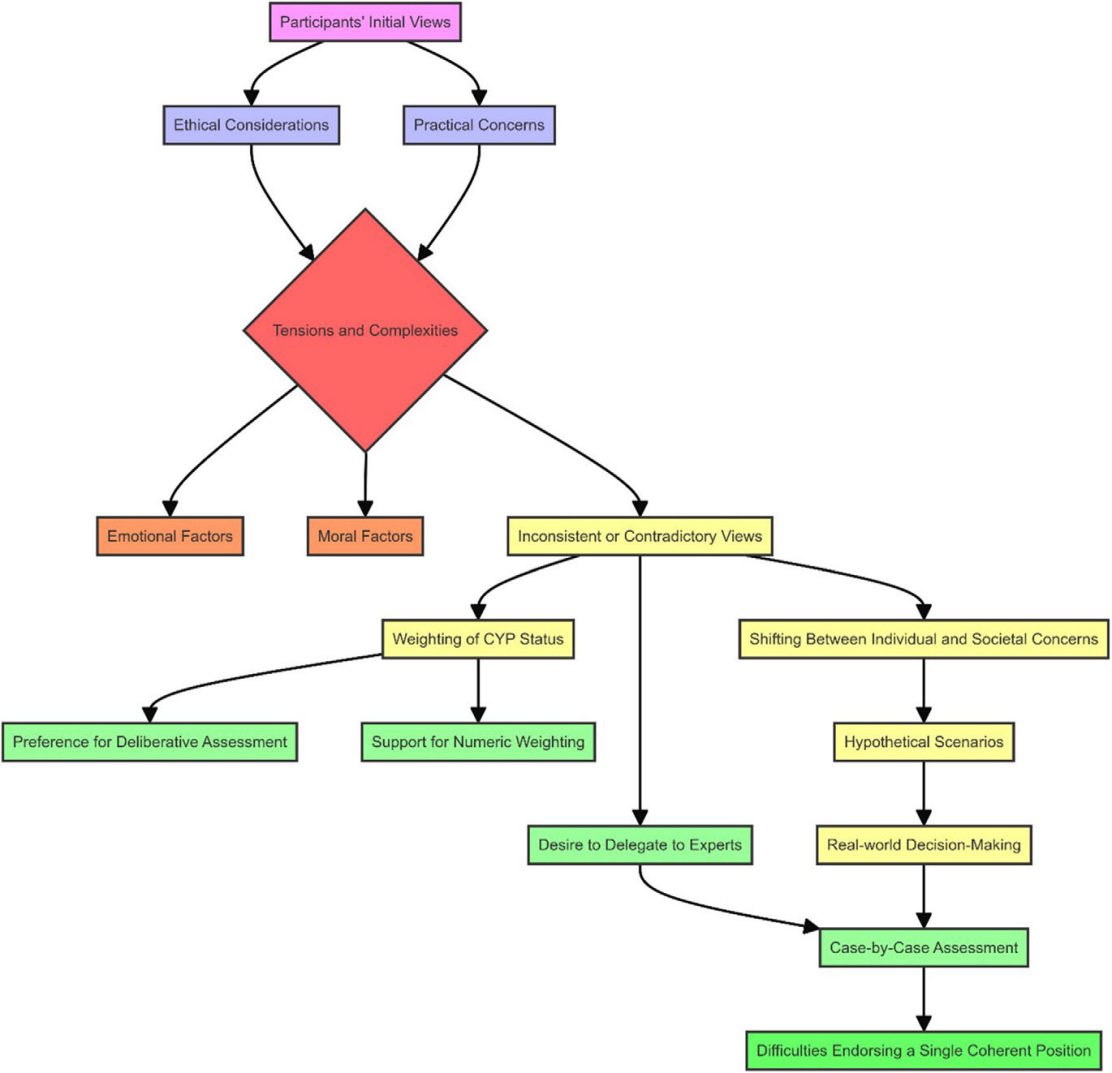
Thematic schema of the inherent tensions developing a coherent or consistent position about the concepts of weighting and the moral relevance of CYP status within the context of HTA decision making.

The themes and their respective subthemes are described in the following paragraphs, commonly shared by both young people and adults unless specified. Examples of participant quotations to illustrate each theme are shown in Table 2. The thematic schema (Figure 2) illustrates the complexities and tensions among themes.

**Table 2.** Illustrative comments by participants and by theme and subtheme

Theme/Subtheme	Participant comments
Inherent tensions in developing a coherent or consistent position	
Difficulty making an informed decision	“I’m mainly coming from an ethical standpoint because I don’t really have the experience in healthcare to understand what the impacts would be by making that change. … I also don’t think that I am qualified to, I don’t have the information, or the expertise to say from a healthcare point of view… people on the panel who make the healthcare decisions are qualified. I could not be on the panel, I’d have no clue what I’m doing…. So I guess that’s mainly why I’m coming from an ethical point of view, because I don’t really have any actual hard facts with me.” (YP07, female, 17, fair health, extensive experience interacting with the healthcare system)
Recognizing inconsistent or contradictory views	“Decision-makers could choose funding using a rule-based thing, and it might actually have a better rational economic outcome. I think it just goes against people’s intentions. So, if you keep it secret, maybe that’s not the worst thing. [Pause] I don’t know. Help me out. [Laughs] …. But then I sort of disagreed with that at the start. That’s so tough, though. Basically, my argument is that children should be treated more than adults, but then … yeah, I don’t really know why. [Laughs].” (A11, male, 20, very good health, minimal experience)
	“It’s very complicated. I usually like treating people exactly equally. But also, equality isn’t the same as equity. I think there are benefits to putting more funding towards young people… But I don’t think age should be the most important factor… Yeah, not sure, sorry. [Age maybe isn’t the most important factor]. But it’s relevant.” (YP05, nonbinary, 16, poor health, extensive experience)
Contrasting hypothetical and real-life decision making	“… if it’s a child who’s going to die if they don’t get the treatment, compared to a 75-year-old, the younger population should be prioritized. But I don’t think something that black and white happens regularly.” (YP02, female, 16, good health, minimal experience)
Weighting of QALYs in HTA submissions	
Negative judgments about numeric weighting: a preference for deliberative, individualized assessment over rigid criteria	“So once again, like, the value of money, I think it can’t just be for the Australian population…my value for money in the medical systems could be way different to yours…I’ll get a lot more out of the mental health field. I have friends who would get a lot more out of the allied health… it really has to be a condition by condition…. value for money… depending on what condition you fall under.” (A07, nonbinary, 18, no children, fair health, extensive experience)
	“… that’s a quick way [use of threshold], sometimes we could do it through a simplistic and quick way, but that may not always be the best way because you still need the human side…. not everything fits into a nice cookie-cutter mould.” (A04, male, 39, no children, very good health, minimal experience)
	“…you could say, “Aboriginal and Torres Strait Islanders get a 1.2 times boost multiplier.” But, surely it depends on how big the decisions are. If it’s a massive decision about funding COVID vaccines … then surely a discussion is the best way to do it. But if, on a smaller level, maybe you could just have a quantitative thing, because that’s gonna get roughly the idea across.” (A11, male, 20, very good health, minimal experience)
Positive judgments about numeric weighting: weighting to support equity and justice	“But to have a decision-maker who is genuinely, as much as a politician can, act with someone else’s best interests at heart, and who goes, “Ah, treat everyone equal but …” you know, maybe don’t spend thousands and thousands of dollars on keeping old people alive for another 3 years before they cark it.” (YP01, female, aged 17, self-reported very good health, with minimal experience)
	“…any submissions for kids should be automatically boosted somewhat? I like that as an idea. Mostly for the financial reasons of families. It’s a bit of a, equality versus equity argument. And so, everyone in society should be treated the same on, a basic, respect everyone level, but different people in society have different needs, and we need to take that into consideration. And so, it falls to their families, and it makes sense that that’s an extra financial burden for the families. And so… it would make sense that they would get an extra boost.” (YP06, male, 17, very good health, minimal experience)
Moral relevance of CYP status in priority-setting	
Lack of endorsement for a universal rule to positively weight health gains in CYP	“I don’t think [kids] are the top group, but there isn’t a lot of healthcare stuff that discriminates other groups more than indigenous people and young people. Lower income has an effect on your health, it definitely does. But probably not to the same extent that being indigenous or being a young person.” (YP10, female, 17, fair health, extensive experience)
	“…blanket rules are never going to work because you can never say “always save the younger person,” because the younger person, you could pour $10,000 into them, and they’ll still die. And your 96-year-old man might have heaps left to give to the world. And I think that looking at as many similar cases as individually as possible is probably going to get you the best results. Because blanket rules just don’t work.” (YP01, Female, aged 17, self-reported very good health, minimal experience)
Other possible priority setting criteria	“…you said something about kids having priority? I think that, while, they should have the priority over just standard, adult treatments or stuff like that, I think it’s probably worth it to prioritize maybe medicine that either can be used for both children and adults, or being able to produce and prioritize the medicine that’s going to be the most effective and the newest advancements before you move onto the kid stuff. So, it’s almost like a ladder or a pyramid.” (Male, 15, good health, minimal experience)
	“…it was frustrating, more kids than you realize have chronic pain conditions but you just don’t really pick it up. Because it doesn’t make sense in an eight-year-old’s body whose body is meant to be healthy because you’re young. So, I think maybe the people who are funding it, think that there’s not many people who have chronic pain conditions so they don’t bother funding it, which is fine. But, there are, so funding would be nice.” (YP07, female, 17, fair health, extensive experience)
	“… I feel like sometimes [the health system is] emergency medicine. We always do band-aid approaches to things rather than preventative approaches [and] don’t take a longer-term approach to fixing things.” (A04, male, 39, no children, very good health, minimal experience)
Governing HTA decision making	
Balancing expertise, representation, and stakeholder preferences	“I think the important thing with decision-making like this is that you’ve got a range of expertise, and you’ve got a range of perspectives presented, that you don’t want this to be purely politicized.” (A03, female, 35, has children, very good health, moderate experience)
	“…there’s two good things that really stand out to me. One is that the committee is independently appointed. There’s no main political drive or bias there, which is good. It’s focusing on the healthcare, the wellbeing of the people who need it. And also, I’m glad that there’s consumer representatives, maybe people who are interested in the drug or intend to take it. It’s like firsthand feedback from a potential consumer. That gives insight into their problems and feedback, which is good.” (A09, male, 18, does not have children, good health, minimal experience)
Navigating transparency	“People should know what’s happening… The public has a right to know, especially when their taxes fund healthcare. Summaries of discussions, key arguments for and against decisions, can keep them informed.” (YP09, male, 16, very good health, minimal experience)
	“What is it that they’re so afraid of the public knowing? If they’re willing to influence decisions that affect all Australians, the Australians being affected should be allowed to know. I don’t know much about commercial pharmaceutical secrets, but I guess that’s an excuse they would use. I think if you didn’t know who the members are, but you knew what they were saying, that’d be great. You knew that X many of them are healthcare professionals and could see what they were saying. But it seems odd to have their names public.” (A17, female, 18, fair health, extensive experience)

### Inherent tensions in developing a coherent or consistent position

Participants struggled to commit to straightforward or coherent positions due to the problem’s complexity. Participants often shifted between individual and social concerns, noted difficulties making an informed decision, or expressed a desire to delegate to experts or rely on case-by-case assessments. Views on health gains for children were sometimes inconsistent, particularly between conceptual and practical considerations or hypothetical versus real-world scenarios. Additionally, participants grappled with incommensurable perspectives and values, considering both short-term and long-term consequences. For these reasons, participants shifted between different positions during the interviews, especially on the concepts of weighting and the moral relevance of CYP status within HTA decision making.

### Weighting and moral relevance of CYP status

**Figure 3.**
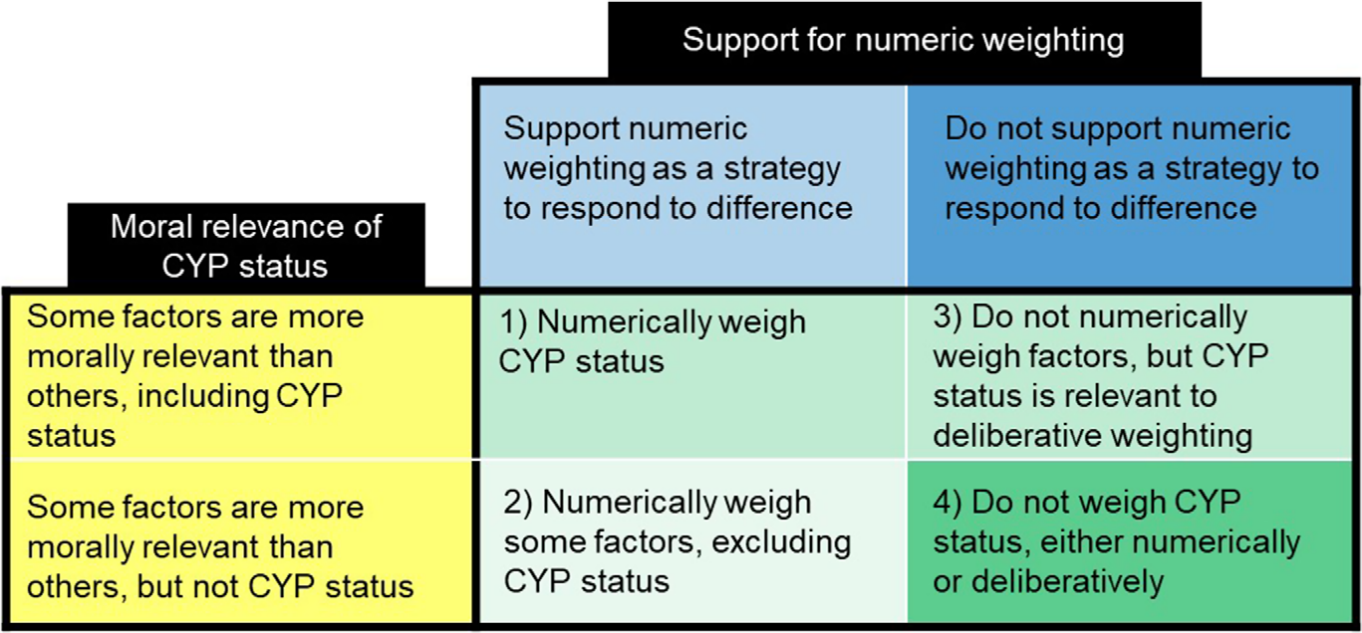
Four positions identified on the value of health gains in relation to weighting and the moral relevance of CYP as a factor in decision making. Although participants sometimes shifted between different positions regarding weighting and the moral relevance of CYP status during the interviews, the darker shades indicate the positions they seemed to most strongly endorse overall.

There appeared to be four main positions available with regard to weighting and the moral relevance of CYP status (Figure 3). A fifth potential position, advocating that “All factors have equal moral relevance and should be weighed equally in HTA decisions,” was not identified in the data.

Almost two thirds of participants opposed the use of numeric weighting, but among those who did not, a majority supported the numeric weighting of CYP status. Additionally, although about half of the participants considered the status of CYP morally relevant and believed it should be considered in decision making, most preferred that this be assessed deliberatively. Due to a pronounced preference against numeric weighting, combined with roughly even levels of disagreement on the moral relevance of CYP status, position 4 (do not weigh CYP status, either numerically or deliberatively) was the most dominant. This stance was closely followed by Positions 1 and 3.

#### Negative judgments about numeric weighting: a preference for deliberative, individualized assessment over rigid criteria

Participants often expressed skepticism about universal measurement of concepts such as value for money, equity, and quality of life across different conditions and life experiences. One concern about numeric weighting was that it would make decision making more reliant on quantitative thresholds or “blanket rules.” Participants worried that this approach could overlook the “human element” of decision making, potentially compromising the overall effectiveness and fairness of healthcare resource allocation. They feared that rule-based prioritization would restrict consideration of contextual factors, leading to inequitable outcomes and failing to account for the different values within various population subgroups and individual needs.

Instead, participants advocated for using evidence about all relevant criteria in each case without assigning predetermined weights to population groups. They argued that each decision should be considered on its merits through a thorough, individualized process.

*I still think it should be a case-by-case application, rather than a blanket rule of “unless you’re 18 you can’t access this,” because there’s always individual circumstances. It should be based on their needs. (A05, female, 48, has children, fair health, extensive experience)*

As an alternative to a universal rule, participants preferred a contextually situated, case-based approach to HTA decision making. Although they acknowledged the limitations and strengths of both rule-based and case-based approaches, some participants suggested that numeric weighting could be used to partially inform deliberative processes or for less consequential decisions, rather than being systematically implemented for population level decisions.

#### Positive judgments about numeric weighting: weighting to support equity and justice

A small number of participants explicitly supported or were open to considering numeric weighting in HTA decision making. They viewed such weighting as a mechanism to address social and economic disparities, believing that systematic weighting of health resources using predefined criteria could promote equity for marginalized groups. However, this belief was nuanced; some felt that efforts to solve inequity should focus on core issues rather than numeric weighting in health decision making. One participant noted that such weighting should be temporary, as it is an overly simplified solution to the broader societal complexities of systemic disadvantage.

### CYP status considered less morally relevant than many other priority-setting criteria

#### Lack of endorsement for a universal rule to positively weight health gains in CYP

Regardless of age, most participants did not endorse a universal rule that CYP’s health gains should have greater value in decision making than health gains in adults. Although many participants expressed a moral intuition that some exceptionalism for CYP’s well-being might be justified, this did not lead them to support a universal or inflexible rule prioritizing CYP’s health gains, especially after considering the potential claims of other groups.

A small group of participants supported giving greater weight to interventions for CYP to promote equity. They cited reasons such as the lack of societal voice for CYP, their disadvantaged or vulnerable status, the need to address health system inequities, and the financial burden on families. However, no participants explicitly identified CYP status as the most important factor for weighting.

#### Other possible priority setting criteria

As participants navigated the complexities of prioritization in HTA, they identified a spectrum of factors that rivalled, and often surpassed, the significance of CYP status. These included both person-centered factors and intervention related factors.

#### Person-centered factors

Participants described a multidimensional “ladder” of need, considering vulnerabilities such as unmet health needs, socioeconomic disadvantages, and systemic inequities. The transient nature of CYP status and concept of intersectionality often prompted a re-evaluation of prioritization of CYP, especially for marginalized groups like Indigenous communities, LGBTQI+ groups, rural/remote communities, and people with disabilities. Notably, Aboriginal and Torres Strait Islander communities were most highlighted for broader healthcare prioritization, seen as having a uniquely compelling claim. In addition, some younger participants suggested prioritizing a sick parent’s health for its broader family impact, emphasizing practical considerations such as feeling safe.

*If the parent can’t work, then they’re kind of screwed. So obviously we don’t want our kids to be sick, that’s awful. But I would much prefer me to be sick than the person who’s providing for me and keeping me safe.* (YP01, female, 17, very good health, minimal experience)

#### Intervention related factors

Participants noted gaps in specialized services for CYP, yet often refrained from endorsing age as the main criterion for healthcare prioritization. They highlighted visible public support for pediatric care, contrasting it with the less pronounced advocacy for adult health care. Despite frustrations in accessing health care, participants did not advocate for systemic prioritization of children but pointed out critical gaps in pain management and mental health support, especially without private insurance.

*Children get a lot of light shone on their issues, which obviously is great. But always on the news you hear it’s about a child … there’s not much helping adults.* (YP04, nonbinary, 16, poor health, minimal experience)

Participants also expressed frustration with modern medicine’s focus on chronic disease management over early intervention and prevention, identifying gaps in public education and resources.

### Governing HTA decision making

Participants emphasized the need for a fair and just system in HTA decision making, identifying two main issues: balancing expertise, representation, and stakeholder preferences and navigating transparency.

#### Balancing expertise, representation, and stakeholder preferences

Participants stressed the importance of diverse representation in HTA committees, combining medical and economic professionals with community voices and those with lived experience, to ensure empathetic and informed decisions. They expressed relief that evaluations were not conducted by politicians or the pharmaceutical industry, but noted that final subsidy decisions rested with the government, preferring independent committee-led decision making. Comparisons to Australia’s COVID-19 response highlighted a distrust of political and industry influence, reinforcing the desire for evidence-based deliberations.

Regarding CYP, participants recognized their often marginalized status and supported their representation on HTA committees. Concerns about CYP’s capacity and maturity were raised, with suggestions for CYP advocates or well-supported representatives to effectively contribute their perspectives.

*In my head, I’m picturing these committees – a really big table, and everyone’s sitting at the table … you can’t just have a nine-year-old at that table, it wouldn’t work. But I do think they have the capacity to be involved in those decisions… if you had people who work with kids, who understand kids, on your team… you could definitely extract some really meaningful opinions or perspectives.* (A18, female, 18, does not have children, fair health, extensive experience)

#### Navigating transparency

Transparency was deemed crucial for public trust. Participants advocated for clear dissemination of procedures, discussions, and decisions, balanced with confidentiality for sensitive details. Suggested solutions included plain English summaries, engaging an independent auditor, and anonymizing decision makers while revealing their qualifications to maintain trust and transparency.

## Discussion

The findings of this study suggest that both young people and adults in Australia acknowledge the moral relevance of CYP status, but opinions differ on its application in HTA. Although some supported numeric weighting, the majority preferred a deliberative, individualized assessment approach. A sizable minority of CYP and adults did not view CYP status as morally relevant. In some cases, CYP or their parents who had experienced a particular condition would express the broad view that CYP were generally well served and did not require weighting within HTA, while emphasizing gaps in some areas of service (e.g., specialist pediatric mental health or pain care). Other factors, especially Aboriginal and Torres Strait Islander status, were often considered more relevant. There was strong support for addressing severity, unmet need, prevention, and early intervention.

Participants often struggled with the inherent tensions between these values, shifting between different positions and struggling to form firm views. This reflects their preference for context-sensitive decision making over rigid numeric weighting, resonating with the flexible, deliberative approach used by most HTA bodies, including the PBAC and MSAC, which allows for a societal perspective to be integrated in the evaluation process (5;8;20). These findings suggest that public views may evolve through structured deliberative exercises, where exposure to different perspectives and trade-offs could lead to more informed and considered positions on CYP in HTA. These findings also align with a UK-based qualitative study, indicating that though the moral relevance of CYP status is recognized, there is limited support for its numeric weighting in HTA decisions (24). However, few qualitative studies have explored societal preferences within HTA processes, limiting cross-country comparisons.

Preference elicitation studies identify nuanced public valuations of health gains (14), but rarely explore explanations for these preferences. A systematic review (22) highlighted variability in preferences for prioritizing children over adults based on question framing and respondent characteristics, with fairness, equity, and spillover effects often underexplored. Our study contributes by examining Australian public views – both adult and young people – within real-world HTA processes, finding little support for numeric weighting of cost-effective thresholds for children.

This research emphasizes the importance of integrating societal values into HTA frameworks, a complex challenge recognized in the literature. There is a significant disconnect in the literature between the theoretical acknowledgment of the importance of societal values and their practical integration into HTA (20;39;40). Expanding qualitative research in this area would improve understanding of how societal values shape decision making across different health systems, addressing this gap.

Structural factors also shape the assessment of pediatric technologies in HTA. The emphasis on evidentiary certainty may disadvantage technologies for children and rare diseases, whereas market-driven review processes may misalign with pediatric needs (41). Although participants were not explicitly given this context, their concerns about unmet needs reflect these broader challenges.

Our results highlight the difficulty of capturing public perspectives on HTA’s complexities and integrating diverse values into decision making. Broad societal consensus may not ensure equitable decisions for specific health interventions, as it may overlook the interplay of effectiveness, public values, and context. Decisions made without considering health system contexts and populations specific needs may not align with the best interests of all stakeholders.

### Strengths and limitations

A strength of this study was the rigorous approach to informing participants, reflected in the depth of their responses. It is the first to engage with young people, a demographic often overlooked in research. Limitations include fewer young participants and higher SEIFA scores compared to the general population. Despite this, no clear difference in perspectives was observed between participants from higher and lower SEIFA areas. Diverse health experiences and online data collection enabled national inclusion (with the exception of the Northern Territory), though social media recruitment undoubtedly influenced the demographic profile of our sample. Nevertheless, this approach also presented advantages, such as the ability to target specific demographics, particularly young males, to balance the gender representation in our sample. Another limitation was that participants were not informed about the age-related implications of QALYs, as the study focused on societal perspectives rather than technical debates regarding QALYs (42).

### Implications for practice/policy

Integrating public preferences into HTA is complex and requires embracing ambiguity and context in policymaking. Our study highlights the importance of involving individuals with lived experience, including younger people, to ensure diverse and relevant perspectives are included. With Australia’s HTA policy framework under review (43), meaningful consumer participation is essential to develop policies that meet technical and economic standards while aligning with societal expectations. Based on our findings, it appears that though CYP status might be considered relevant in deliberations on some technologies, there was little support among our participants for a system of predetermined numerical weights for population characteristics in HTA decision making.

### Implications for research

Our findings suggest future research directions. First, explore under-represented SEIFA groups to understand their perspectives. Second, broaden research to include valuation and HTA issues with the general public and international decision makers, beyond CYP. Qualitative studies in countries using numeric weighting in HTA could identify how decision makers apply or deviate from these methods. Finally, given our success in engaging young people on HTA issues, continue researching CYP’s perspectives on complex healthcare issues, such as accessing specialist services in Australia (e.g., mental health, pain medicine).

## Conclusion

Australian young people and adults hold a nuanced appreciation for integrating societal values in HTA. Although most participants recognized some moral relevance of CYP status in HTA, opinions varied on its practical application. A minority supported numeric weighting of CYP status, whereas a larger group advocated for its reflection through individualized, deliberative assessments. Participants often considered other factors more relevant, such as severity and unmet needs, especially for Aboriginal and Torres Strait Islander communities. The varied viewpoints and difficulty in forming concrete positions of incorporating societal values in HTA suggest that current decision-making approaches without numeric weights for CYP align broadly with the perspectives of our study participants.

## Supporting information

Sellars et al. supplementary material Sellars et al. supplementary material

## Supplementary material

The supplementary material for this article can be found at http://doi.org/10.1017/S0266462325000194.
