# Supplementary material for: “Blanket rules just don’t work”: Qualitative exploration of the relative value of child and adult quality-adjusted life year (QALY) gains for health technology assessment
Source: Int J Technol Assess Health Care. 2025 Mar 28;41(1):e23. doi: 10.1017/S0266462325000194 (PMC12019764; doi:10.1017/S0266462325000194)
Supplement: Sellars et al. supplementary material [file S0266462325000194sup001.docx]

**Supplementary Materials**

**Online Supporting Information 1**. Interview guide.

| **CHECKLIST** | * RECORDING * CONFIDENTIALITY & ANONYMITY * STOPPING * TRANSCRIPTS  * CONSENT TO PARTICIPATE [record oral consent as per form] | 1. **Health and care in children and adults** | - How do you feel about prioritizing healthcare for children compared to adults?  - Do you see any benefits or problems with this approach? Why or why not?  - Do you think it is important to think about age when making decisions about approving treatments to be funded? Why or why not?  - (E.g. equal opportunities to live a full life, investing in future, potential productivity benefits, etc?) |
| --- | --- | --- | --- |
| 1. **Experience accessing health and care services** | - Can you briefly describe any experiences you’ve had with healthcare services?  - Would you say you have had a lot, a moderate amount, or little experience accessing healthcare services? - Have you accessed services for yourself and/or for family members? How do these experiences compare?  - Have you or someone you know ever been unable to access a specific healthcare service, treatment, or medication? (This could be a personal experience, something you heard from someone, or something seen in the media.)  - If so, how did that experience make you feel? Do you think it was justified? Why or why not? | 1. **Social values / attributes** | **[Interviewer to present arguments from pre-interview survey]**  - Based on the arguments in the pre-interview survey, did you agree/disagree with these arguments? Why or why not?  - How do you feel that the government sometimes uses reasons like this to decide about whether to fund treatments? |
| 1. **Awareness and initial reactions to health technology assessment** | - Have you heard of the process used to assess and approve funding for new health technologies? - How do you feel about this process? Do you think it should exist? Why or why not?  - In general, how do you think decisions about funding new drugs and health care should be made?  **[Interviewer to read aloud broad description of how decisions are made to fund new health technologies]**  - After hearing this description of funding decisions are made, do you agree with this approach? Why or why not? - Do you think we should focus more attention on developing new drugs or health care initiatives for some groups more than others (e.g. younger versus older individuals, lower income, certain diseases)? Why or why not? | 1. **Close** | - Is there anything you would like to add about what we have discussed today?  - Does anything else come to mind that you think is important to consider?  **Thank you very much for taking part in this interview.** |

| **Online Supporting Information 2.** Pre-interview survey. |
| --- |
| **Section 1.** Governments have a limited budget for healthcare and cannot afford to provide everything. They have to make decisions about whether to fund drugs, treatment and health services for children and adults. They often do this based on whether the treatments are “value for money”.  Value is about improving health. So, treatments that give a lot of improvement in health for, say, $1000, would have greater “value for money” than treatments that only give a small improvement in health for the same amount of money.  When making decisions, healthcare that gives greater “value for money” is prioritized. Healthcare that doesn’t give as much “value for money” may not be provided.  Some people say that healthcare for children should be a higher priority than healthcare for adults. This is the same as saying that healthcare for children could be funded even if it did not give as much “value for money” as healthcare for adults.  What are your initial thoughts about this? |
| **Section 2.** Now I want to tell you why some people say that healthcare for children and young people should be funded even when it gives less “value for money” than healthcare for adults.  - Children and young people should have the chance to grow up and experience all stages of life.  - Children and young people will work and contribute to society for longer than adults.  - Children and young people often don’t get as much of a say as adults in decisions about funding, so they should get an “extra boost” to make sure things are fair  - When children and younger people benefit, their family benefits too  Do you have any thoughts about the reasons listed above? Do you agree or disagree with any of them?  Remember, there are no right or wrong answers and we will talk more about this in your interview. |
| **Section 3.** Okay, now on the other hand, I want to tell you why some people say that healthcare for children and young people should not be funded when it offers less “value for money” than healthcare for adults.  - Everyone in society should be treated the same: they should have the same access to treatments no matter how young or old they are  - Adults have worked and contributed to society for longer, so it is not fair to treat them differently to children and young people  - If we have to give an “extra boost” to some people in society, it should not be because they are young or old. There are other more important things  Again, do you have any thoughts about the reasons listed above (e.g. do you agree or disagree with any of them)?  Remember, there are no right or wrong answers and we will talk more about this in your interview. |
